# Clinical and Nonclinical Factors and Advanced Neonatal Resuscitation Interventions

**DOI:** 10.1001/jamanetworkopen.2026.9923

**Published:** 2026-04-30

**Authors:** Breanna Pickett, Bo Pan, Susan Crawford, Georg M. Schmölzer, Deborah A. McNeil, Amuchou Singh Soraisham, Heather Shonoski, Khalid Aziz, Brenda Hiu Yan Law

**Affiliations:** 1 Department of Pediatrics, Faculty of Medicine and Dentistry, University of Alberta, Edmonton, Alberta, Canada; 2 Epidemiology Coordinating and Research (EPICORE) Centre, Department of Medicine, Faculty of Medicine and Dentistry, University of Alberta, Edmonton, Alberta, Canada; 3 Alberta Health Services, Alberta, Canada; 4 Centre for the Studies of Asphyxia and Resuscitation, Neonatal Research Unit, Royal Alexandra Hospital, Edmonton, Alberta, Canada; 5 Department of Pediatrics, Cumming School of Medicine, University of Calgary, Calgary, Alberta, Canada; 6 Department of Family Medicine, Faculty of Medicine and Dentistry, University of Alberta, Edmonton, Alberta, Canada

## Abstract

**Importance:**

Approximately 1% of newborns receive advanced neonatal resuscitation interventions (ANRIs; consisting of endotracheal intubation, chest compressions, and epinephrine administration), the likelihood of which increases with known clinical factors. Associations of socioeconomic status (SES) and health system factors with ANRI are unknown.

**Objective:**

To examine the associations among maternal SES, remoteness of maternal residence, birth-site level of service, clinical factors, and ANRIs.

**Design, Setting, and Participants:**

This cross-sectional study used an administrative dataset of all live births at 34 weeks’ gestation or later between January 1, 2000, and December 31, 2020, in Alberta, Canada, a jurisdiction with universal health care and provincially coordinated Neonatal Resuscitation Program training. Hospital births and home births attended by registered midwives were included. Statistical analyses were conducted from August 1 to September 30, 2025.

**Exposures:**

Maternal SES (classified according to Pampalon Material Deprivation Index quintiles), remoteness of residence (categorized using the Statistics Canada Remoteness Index), birth-site level of service, and clinical factors (including maternal, intrapartum, and fetal or neonatal characteristics).

**Main Outcomes and Measures:**

The primary outcome was any ANRI. Secondary outcomes were individual interventions (endotracheal intubation, chest compression, epinephrine administration). Multivariable logistic regression with stepwise variable selection was used to examine associations between SES and health system factors and the primary outcome.

**Results:**

A total of 966 475 live births at 34 weeks’ gestation or later were analyzed, of whom 1.0% received ANRI. Neonates had a mean (SD) gestational age of 38.9 (1.48) weeks and included 494 835 males (51.2%); mothers had a mean (SD) age of 29.4 (5.46) years. Compared with level 2 birth sites, level 3 birth sites had lower odds of ANRI (odds ratio [OR], 0.57; 95% CI, 0.53-0.61) and all interventions. Births at level 1A sites (OR, 2.53; 95% CI, 1.99-3.15) and home births (OR, 1.44; 95% CI, 1.18-1.74) had higher odds of ANRI and chest compressions (OR, 7.32 [95% CI, 5.61-9.40] and 4.12 [95% CI, 3.29-5.11], respectively). All level 1 births (level 1C: OR, 0.54; 95% CI, 0.49-0.60; level 1B: OR, 0.52; 95% CI, 0.45-0.59; and level 1A: OR, 0.4 95% CI, 0.20-0.71) and home births (OR, 0.27; 95% CI, 0.16-0.43) were associated with lower odds of endotracheal intubations. There were no associations for maternal SES and maternal residence remoteness. The most significant clinical factors associated with ANRI were maternal general anesthesia (OR, 4.89; 95% CI, 4.47-5.34), lower gestational age (highest OR at 34 weeks: 3.60; 95% CI, 3.11-4.15), cesarean delivery (OR, 1.80; 95% CI, 1.60-2.02), meconium-stained amniotic fluid (OR, 2.05; 95% CI, 1.94-2.17), and macrosomia (OR, 1.83; 95% CI, 1.55-2.16).

**Conclusions and Relevance:**

In this cross-sectional study, maternal SES and maternal residence remoteness were not associated with increased odds of ANRI, and birth sites with a lower level of service were associated with higher odds of chest compressions but lower odds of endotracheal intubations. Clinicians should be prepared to resuscitate neonates exposed to general anesthesia.

## Key Points

**Question:**

For late preterm and term newborns, are nonclinical factors—maternal socioeconomic status (SES), remoteness of residence, and birth-site level of service—associated with odds of advanced neonatal resuscitation interventions (ANRIs)?

**Findings:**

In this cross-sectional study of nearly 1 million births, maternal SES and maternal residence remoteness were not associated with higher odds of receiving ANRI. Birth sites with lower levels of service were associated with higher odds of chest compressions.

**Meaning:**

The findings of this study highlight a need to improve neonatal resuscitation in settings with lower levels of service.

## Teaser

This cross-sectional study evaluates the association of maternal socioeconomic status, remoteness of residence, and level of service at delivery settings with use of advanced neonatal resuscitation interventions in late preterm and term newborns.

## Introduction

Approximately 10% of newborns receive resuscitation at birth, with 1% receiving advanced neonatal resuscitation interventions (ANRIs), which include endotracheal intubation, chest compressions, and/or epinephrine administration.^1,2^ The risk for ANRI increases with clinical factors, such as preterm birth, maternal disease, and low birth weight.^3^ Neonatal resuscitation may also be impacted by socioeconomic status (SES) and health system factors, such as maternal SES, geographical remoteness, and health services availability.

Direct measures of SES are difficult to obtain; thus, proxy measures have been developed. Pampalon et al^4,5^ generated indices for social and material deprivation in Canada using 6 Census-based socioeconomic indicators for populations (approximately 400-700 people) in geographical units called dissemination areas. The Material Deprivation Index (MDI) has been used to study SES and pregnancy outcomes.^6^ Geographical care access may also contribute to health disparities.^7^ Thus, in 2017, Statistics Canada developed a Remoteness Index (RI) based on dissemination areas to explore disparities between urban, rural, and remote populations.^8^

Birthing sites have different access to services, including specialists, cesarean delivery, and neonatal intensive care units (NICUs). These capabilities can be categorized into level of service, which may impact perinatal outcomes.^9^ While tertiary centers often receive high-risk pregnancies for delivery and possible resuscitation, many deliveries occur in community hospitals or even at home.^10,11,12^ Educational programs, such as the Neonatal Resuscitation Program (NRP), have thus been created as an attempt to standardize practices across sites based on current resuscitation guidelines.^13,14,15,16,17,18^ Fortunately, risk of resuscitation decreases with increasing gestational age; ANRIs are rarely performed for neonates born at 34 weeks’ gestation or later.^19^ However, community health care practitioners taking care of this population may still need to perform ANRIs, while having less experience and resources than tertiary teams.^9,20^

In our previous univariate analysis of approximately 1 million late preterm and term births in Alberta, Canada, we found higher odds of chest compressions (odds ratios [ORs] ranging from 1.55 to 4.50) and lower odds of endotracheal intubations (ORs ranging from 0.14 to 0.60) in home births and births at level-1A or level-1B sites, highlighting the need for a joint effects model to account for clinical and other nonclinical factors.^2^ Specifically, there is limited research on the associations of SES and health system factors with ANRIs in high-income countries such as Canada, particularly for late preterm and term neonates and in community hospitals. Therefore, using this large dataset, we aimed to examine the associations among maternal SES, remoteness of maternal residence, birth-site level of service, clinical factors, and ANRIs.

## Methods

This retrospective, population-based cross-sectional study included all live births at 34 weeks’ gestation or later in Alberta between January 1, 2000, and December 31, 2020, encompassing the third to the seventh editions of the NRP’s *Textbook of Neonatal Resuscitation*.^13,14,15,16,17^ The University of Alberta Research Ethics Board approved the study and waived the informed consent requirement as individual consent was not possible for the retrospective use of a population-wide dataset. We followed the Strengthening the Reporting of Observational Studies in Epidemiology (STROBE) reporting guideline for cross-sectional studies.

### Dataset, Outcomes, and Covariates

We used the Alberta Perinatal Health Program PeriLink database, which collected data for all hospital births, home births attended by registered midwives, and neonatal deaths.^21^ The neonatal resuscitation interventions recorded included suction, free-flow oxygen, mask ventilation, endotracheal intubation for meconium suctioning, intubation for ventilation, chest compressions, epinephrine, naloxone, and other medications; each had a *yes* indicated if it was provided. No interventions and interventions unknown were indicated separately.

The primary outcome was any ANRI, which was coded as yes if intubation for ventilation, chest compressions, or epinephrine was listed as yes. Clinical factors that may affect the need for resuscitation were collected. Dichotomous fields coded as yes or were left blank were collected. Categorial fields had *unknown* and *no* coded explicitly, such as meconium-stained amniotic fluid (MSAF; recorded as thin, thick, unknown, or none). The dataset abstracted from the PeriLink database was linked with the Alberta Health Services Analytics Discharge Abstract Database (which collects outcomes based on Canadian Institute for Health Information standards^22^) and the Alberta Congenital Anomalies Surveillance System (which records congenital anomalies identified before age 1 year).^23^

All 97 Alberta hospitals that recorded a live birth within the study period were contacted to verify their annual level of service, based on Alberta Obstetrical Triage Acuity Scale, for availability of delivery support, cesarean delivery, obstetricians, pediatricians or neonatologists, and NICUs.^24^ Two categories were added: planned midwife-assisted home birth and unplanned out-of-hospital births (Table 1).

**Table 1.** Clinical and Nonclinical Variables Considered in the Model

Variable	Type	Description
Nonclinical factors		
Maternal SES: Pampalon MDI	Categorical	5 MDI quintiles: least deprived, less deprived, moderately deprived, more deprived, most deprived
Maternal residence RI	Categorical	5 Categories based on k-means clustering of RI using maternal postal codes: easily accessible area, accessible area, less accessible area, remote area, very remote area
Birth-site level of service	Categorical	Level 0: no obstetrical services, no planned deliveries
		Level 1A: planned deliveries, but no operating room capability
		Level 1B: 24/7 operating room or cesarean delivery capability
		Level 1C: operating room or cesarean delivery capability and obstetricians
		Level 2: obstetrics and pediatric services including level 2 NICU
		Level 3: obstetrics and pediatric services including level 3 (highest level) NICU
		Planned midwife-assisted home birth: planned midwife-attended birth at home or at another nonhospital location
		Unplanned OOH births: births occurring before arrival at hospital or before arrival of midwife
Clinical factors: maternal		
Maternal age, y	Continuous	Maternal age in y
Maternal age <20 y	Binary	NA
Maternal age >35 y	Binary	NA
Maternal age categories	Categorical	Risk-based categories based on maternal age: <20 y, 20-35 y, >35 y
Maternal heart disease: asymptomatic	Binary	NA
Maternal heart disease: symptomatic	Binary	NA
Previous cesarean delivery	Binary	NA
Preexisting hypertension	Binary	NA
Gestational hypertension	Binary	NA
Prepregnancy weight >91 kg	Binary	NA
Preexisting diabetes	Binary	NA
Gestational diabetes	Binary	NA
Clinical factors: intrapartum		NA
Cesarean delivery: elective	Binary	NA
Cesarean delivery: emergency	Binary	NA
Cesarean delivery: any type	Binary	NA
Induction of labor	Binary	NA
Type of labor	Categorical	4 Categories: spontaneous, induced, none, unknown or missing
Epidural anesthesia	Binary	NA
Spinal anesthesia	Binary	NA
General anesthesia	Binary	NA
Augmentation of labor	Binary	NA
Forceps or vacuum-assisted labor	Binary	NA
MSAF	Binary	NA
Clinical factors: fetal or neonatal		
GA at delivery	Categorical	GA in completed wk: 34-45
Preterm	Binary	GA <37 completed wk
Birth weight	Continuous	Weight at birth in kg
Low birth weight	Binary	<2.5 kg
SGA	Binary	<10 Percentile birth weight based on Fenton growth charts
LGA	Binary	>90 Percentile birth weight based on Fenton growth charts
Extreme LGA	Binary	>97 Percentile birth weight based on Fenton growth charts
Macrosomia	Binary	>4 kg Birth weight
Multiple births	Binary	NA
Congenital anomaly	Binary	Based on Alberta Congenital Anomalies Surveillance System

Abbreviations: GA, gestational age; LGA, large for gestational age; MDI, Material Deprivation Index; MSAF, meconium-stained amniotic fluid; NA, not applicable; NICU, neonatal intensive care unit; OOH, out of hospital; RI, Remoteness Index; SES, socioeconomic status; SGA, small for gestational age.

Two additional nonclinical variables were examined. Maternal SES was quantified via the Pampalon MDI quintiles using maternal postal codes. The Pampalon MDI was generated from 6 indicators selected due to their known links with health, previous use as geographical proxies, availability in Canadian Census data, and affinity with material or social deprivation. Three indicators for people 15 years or older are incorporated into the MDI: proportion without high school diploma, the employment to population ratio, and mean income.^5,25^ The Statistics Canada RI determines the distance from each community to population centers and then normalizes the values from 0 to 1.^8^ Maternal residence RI was categorized via the k-means clustering classification of the RI value, which was assigned using maternal postal codes. This classification was selected because it considers natural data breaks.^8^

### Statistical Analysis

Analyses were conducted from August 1 to September 30, 2025, using R 3.4.0 (R Project for Statistical Computing) and SAS 9.4 (SAS Institute Inc) following an as-observed principle. Preliminary screening verified birth dates within the study period, and variables were selected based on clinical relevance and completeness. Categorical variables were analyzed using χ^2^ or Fisher exact tests, while continuous variables were assessed with unpaired, 2-tailed *t* tests or Wilcoxon rank sum tests, as dictated by data distribution. Regression was performed to evaluate associations of SES and health system factors with any ANRI (the primary outcome), with all statistical assumptions rigorously verified.

Logistic regression analyses were performed at univariable and multivariable levels, with the multivariable models informed by univariable analyses. Results are presented as ORs with corresponding 95% CIs and *P* values. For SES and health system factors, least deprived quintile and easily accessible areas were chosen as reference categories, reflecting optimal care. Level 2 served as the reference for birth-site level of service, as these sites have access to level 2 NICUs but are not anticipated to receive the highest-risk pregnancies for delivery. References for clinical variables were guided by the existing literature. Univariable regression was used to assess each factor’s individual implications for the primary outcome, by fitting models for each factor separately, whereas multivariable regression was fitted to quantify the joint implications of a selected set of factors simultaneously, accounting for correlations among them. Variables included in the multivariable regression were selected based on clinical significance, statistical significance, or relevance to research objectives. Clinical significance was determined by research team expertise, while statistical significance was informed by univariable analyses and stepwise selection using bidirectional elimination to identify a champion model with the optimal set of factors that best fit the data. Two-sided *P* < .05 indicated statistical significance. These factors were subsequently applied as dependent variables in multivariable regression analyses for the secondary outcomes (endotracheal intubation, chest compressions, and epinephrine administration).

We conducted sensitivity analyses to assess the potential implications of missing data for any ANRI. Logistic regression models were fitted to the observations with missing outcomes (resuscitation measures unknown) and the completed dataset in which missing values were imputed, and results were compared with the primary analysis. Imputation was performed to handle missing data.^26,27,28^

## Results

In total, 966 475 live births at 34 weeks’ gestation or later were included, of whom 4928 (0.5%) were excluded for missing birth dates. Neonates consisted of 471 640 females (48.8%) and 494 835 males (51.2%) with a mean (SD) gestational age of 38.9 (1.48) weeks. Mothers had a mean (SD) age of 29.4 (5.46) years. Of the 966 475 neonates, 9202 (1.0%) received any ANRI, 7667 (0.8%) received intubation for positive-pressure ventilation, 2399 (0.2%) received chest compressions, and 165 (0.02%) received epinephrine administration. For 7.4% of neonates, resuscitation measures were listed as unknown in the data source. A description of this population and resuscitation patterns was previously published.^2^

Most births occurred in hospitals with level 2 (56.6%) or level 3 (21.5%) NICUs (Table 2). Births were evenly distributed across MDI quintiles. Most mothers resided in easily accessible (72.7%) or accessible (18.7%) areas, with decreasing numbers from less accessible (5.9%), remote (1.6%), and very remote areas (0.2% [n = 1510]). There was a low effect size, direct correlation between maternal residence remoteness and deprivation (Spearman correlation coefficient [*r*] = 0.17; 95% CI, 0.17-0.17), a medium effect size, inverse correlation between birth-site level of service and maternal residence remoteness (*r* = −0.43; 95% CI, −0.44 to −0.42), and no association between hospital LOS and deprivation (*r* = −0.09; 95% CI, −0.01 to −0.09).

**Table 2.** Final Model for ANRI After Multivariable Regression With Stepwise Variable Selection

Variable and category	No. (%)	OR (95% CI)	*P* value
MDI quintile			
Least deprived	165 422 (17.1)	1 [Reference]	NA
Less deprived	181 015 (18.7)	1.12 (1.04-1.21)	.002
Moderately deprived	178 658 (18.5)	1.04 (0.97-1.13)	.27
More deprived	178 003 (18.4)	1.04 (0.96-1.12)	.30
Most deprived	214 600 (22.2)	0.95 (0.88-1.02)	.17
Maternal residence RI			
Easily accessible area	702 274 (72.7)	1 [Reference]	NA
Accessible area	180 277 (18.7)	0.78 (0.73-0.83)	<.001
Less accessible area	57 066 (5.9)	1.30 (1.18-1.43)	<.001
Remote area	15 406 (1.6)	0.79 (0.63-0.99)	.045
Very remote area	1510 (0.2)	0.65 (0.20-1.55)	.39
Birth-site level of service			
Level 2	547 426 (56.6)	1 [Reference]	NA
Level 0	1307 (0.1)	1.37 (0.71-2.37)	.31
Level 1A	7690 (0.8)	2.53 (1.99-3.15)	<.001
Level 1B	93 735 (9.7)	0.69 (0.62-0.77)	<.001
Level 1C	93 585 (9.7)	0.61 (0.56-0.66)	<.001
Level 3	208 264 (21.5)	0.57 (0.53-0.61)	<.001
Unplanned OOH birth	276 (0)	2.25 (0.55-5.93)	.16
Planned midwife-assisted home birth	14 182 (1.5)	1.44 (1.18-1.74)	<.001
Maternal factors			
Maternal age, y			
<20	37 464 (3.9)	1.32 (1.18-1.47)	<.001
20-35	800 996 (82.9)	1 [Reference]	NA
>35	128 015 (13.2)	0.99 (0.93-1.06)	.82
Preexisting hypertension	8924 (0.9)	1.40 (1.18-1.65)	<.001
Gestational hypertension	52 437 (5.4)	1.27 (1.17-1.37)	<.001
Prepregnancy weight >91 kg	88 039 (9.1)	1.19 (1.10-1.27)	<.001
Preexisting diabetes	10 911 (1.1)	1.31 (1.17-1.54)	<.001
Previous cesarean delivery	139 199 (14.4)	0.78 (0.72-0.84)	<.001
Intrapartum factors			
Cesarean delivery			
Any type	264 252 (27.3)	1.80 (1.60-2.02)	<.001
Elective	142 892 (14.8)	0.78 (0.69-0.89)	<.001
Emergency	87 731 (9.1)	1.45 (1.30-1.62)	<.001
Forceps or vacuum-assisted labor	115 168 (11.9)	1.52 (1.41-1.63)	<.001
Augmentation of labor	419 307 (43.4)	0.92 (0.87-0.97)	<.001
Type of labor			
Spontaneous	541 342 (56.0)	1 [Reference]	NA
Induced	290 076 (30.0)	0.96 (0.91-1.02)	.19
None	117 960 (12.2)	0.89 (0.80-0.98)	.02
Unknown or missing	17 097 (1.8)	0.65 (0.53-0.79)	<.001
Maternal anesthesia			
Epidural	290 856 (30.1)	1.82 (1.72-1.91)	<.001
Spinal	89 066 (9.2)	1.39 (1.27-1.51)	<.001
General	13 350 (1.4)	4.89 (4.47-5.34)	<.001
MSAF	111 535 (11.5)	2.05 (1.94-2.17)	<.001
Fetal or neonatal factors			
GA at birth, wk			
40	246 022 (25.5)	NA	NA
34	10 682 (1.1)	3.60 (3.11-4.15)	<.001
35	16 991 (1.8)	2.49 (2.18-2.85)	<.001
36	34 743 (3.6)	1.70 (1.51-1.91)	<.001
37	78 805 (8.2)	1.39 (1.26-1.53)	<.001
38	181 418 (18.8)	1.02 (0.94-1.10)	.66
39	273 103 (28.3)	1.02 (0.95-1.09)	.58
41	121 124 (12.5)	1.12 (1.04-1.22)	.004
42	3479 (0.4)	1.58 (1.11-2.17)	.007
43	90 (0)	3.19 (0.51-10.71)	.12
44	16 (0)	0.00 (NA-0.40)	0.91
45	2 (0)	0.00 (NA->999.99)	0.96
Multiple births	26 057 (2.7)	0.79 (0.69-0.89)	<.001
Birth weight, per 1-kg increase	NA	0.82 (0.78-0.87)	<.001
Macrosomia	14 771 (1.5)	1.83 (1.55-2.16)	<.001

Abbreviations: ANRI, advanced neonatal resuscitation intervention; GA, gestational age; MDI, Material Deprivation Index; MSAF, meconium-stained amniotic fluid; NA, not applicable; OOH, out of hospital; OR, odds ratio; RI, Remoteness Index.

**Figure 1.**
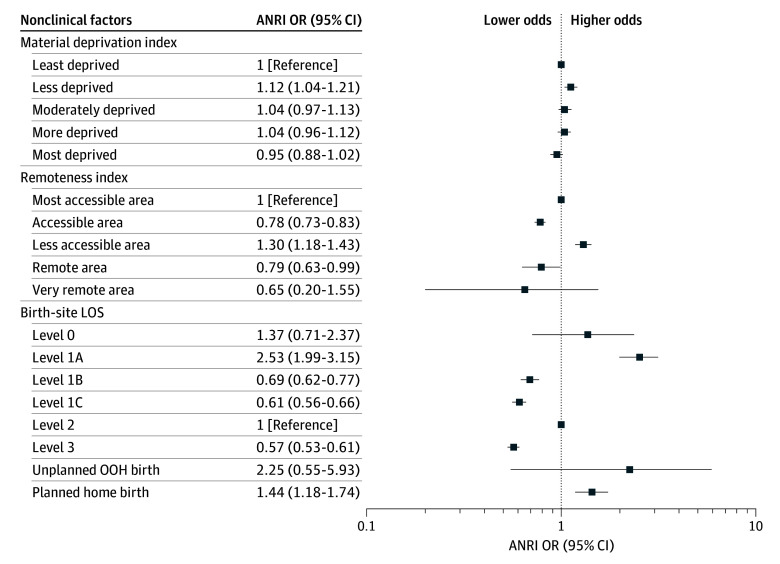
Dot Plot of Socioeconomic and Health System Factors and Odds of Advanced Neonatal Resuscitation Intervention (ANRI) Error bars represent 95% CIs. LOS indicates level of service; OOH, out of hospital; and OR, odds ratio.

**Figure 2.**
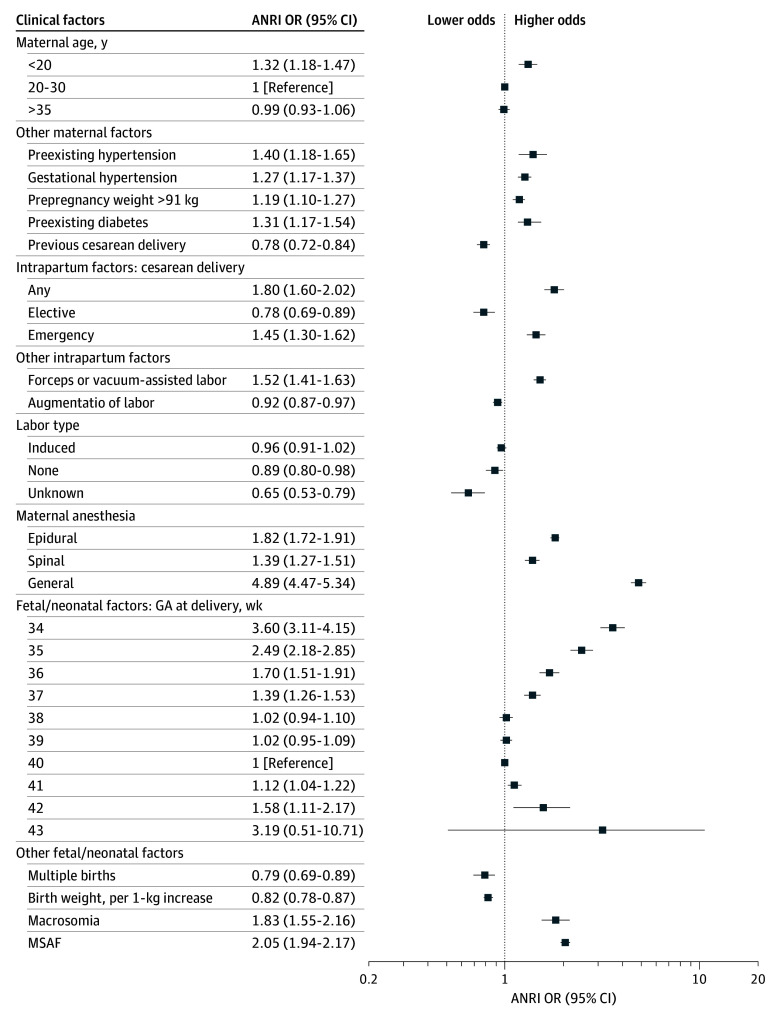
Dot Plot of Clinical Factors and Odds of Advanced Neonatal Resuscitation Intervention (ANRI) Error bars represent 95% CIs. GA indicates gestational age; MSAF, meconium-stained amniotic fluid; OR indicates odds ratio.

Univariable logistic regression was performed on 36 variables; the results and reasons for exclusion in subsequent analyses are provided in eTable 1 in Supplement 1. Next, 32 variables were included in the multivariable logistic regression. After stepwise variable selection, 23 variables remained; multivariable ORs with 95% CIs and *P* values for any ANRI are shown in Table 2, Figure 1, and Figure 2. Several neonatal variables (small, large, and extremely large for gestational age and congenital anomalies) were removed as they did not contribute to best model performance. Two maternal variables (asymptomatic heart disease and gestational diabetes) were not statistically significant and removed. Multivariable logistic regression was conducted for each intervention using the same 23 variables (Table 3; eFigure in Supplement 1). Additionally, we performed an analysis of collinearity with variance inflation factor (VIF) scores for variables in the final model. All VIF scores were lower than 3, indicating that multicollinearity was not a concern.

**Table 3.** Secondary Outcomes: Endotracheal Intubation, Chest Compressions, Epinephrine Administration

Variable and category	Multivariable OR (95% CI)		
	Endotracheal intubation	Chest compressions	Epinephrine administration
MDI quintile			
Least deprived	1 [Reference]	1 [Reference]	1 [Reference]
Less deprived	1.13 (1.04-1.22)^a^	1.09 (0.94-1.27)	0.68 (0.38-1.21)
Moderately deprived	1.04 (0.96-1.13)	1.05 (0.90-1.21)	0.79 (0.45-1.38)
More deprived	1.02 (0.94-1.1)	1.09 (0.94-1.27)	0.76 (0.44-1.33)
Most deprived	0.94 (0.86-1.02)	1.02 (0.88-1.19)	0.75 (0.44-1.30)
Maternal residence RI			
Easily accessible area	1 [Reference]	1 [Reference]	1 [Reference]
Accessible area	0.76 (0.71-0.82)^b^	0.90 (0.80-1.01)	1.10 (0.69-1.70)
Less accessible area	1.45 (1.30-1.61)^a^	0.94 (0.78-1.13)	0.89 (0.40-1.78)
Remote area	0.78 (0.58-1.02)	0.75 (0.53-1.05)	0.36 (0.02-1.78)
Very remote area	0.99 (0.30-2.40)	0.30 (0.02-1.35)	3.99 (0.20-23.46)
Birth-site level of service			
Level 2	1 [Reference]	1 [Reference]	1 [Reference]
Level 0	1.08 (0.46-2.10)	2.86 (1.13-5.90)^a^	13.46 (0.74-66.63)
Level 1A	0.40 (0.20-0.71)^b^	7.32 (5.61-9.40)^a^	0
Level 1B	0.52 (0.45-0.59)^b^	1.55 (1.31-1.82)^a^	1.64 (0.87-2.96)
Level 1C	0.54 (0.49-0.60)^b^	0.92 (0.79-1.06)	1.09 (0.60-1.87)
Level 3	0.64 (0.60-0.68)^b^	0.47 (0.41-0.54)^b^	0.59 (0.36-0.95)^b^
Unplanned OOH birth	0.99 (0.06-4.39)	4.62 (0.76-14.50)	0
Planned midwife-assisted home birth	0.27 (0.16-0.43)^b^	4.12 (3.29-5.11)^a^	1.15 (0.06-5.63)
Maternal factors			
Maternal age, y			
<20	1.20 (1.06-1.36)^a^	1.56 (1.29-1.87)^a^	1.36 (0.60-2.67)
20-35	1 [Reference]	1 [Reference]	1 [Reference]
>35	1.02 (0.95-1.10)	0.88 (0.76-1.01)	0.74 (0.40-1.25)
Preexisting hypertension	1.44 (1.21-1.70)^a^	0.99 (0.64-1.46)	2.29 (0.68-5.78)
Gestational hypertension	1.27 (1.16-1.38)^a^	1.29 (1.08-1.53)^a^	1.11 (0.56-2.03)
Prepregnancy weight >91 kg	1.21 (1.12-1.31)^a^	1.05 (0.91-1.22)	1.20 (0.68-1.98)
Preexisting diabetes	1.27 (1.07-1.50)^a^	1.31 (0.90-1.83)	1.39 (0.33-3.85)
Previous cesarean delivery	0.78 (0.72-0.85)^b^	0.69 (0.58-0.82)^b^	0.25 (0.12-0.48)^b^
Intrapartum factors			
Cesarean delivery			
Any type	1.94 (1.71-2.19)^a^	1.77 (1.41-2.20)^a^	5.51 (2.51-11.65)^a^
Elective	0.79 (0.69-0.90)^b^	0.69 (0.54-0.87)^b^	0.56 (0.27-1.18)
Emergency	1.56 (1.39-1.76)^a^	1.06 (0.86-1.32)	0.94 (0.52-1.79)
Forceps or vacuum-assisted labor	1.75 (1.61-1.89)^a^	1.02 (0.87-1.19)	4.35 (2.37-7.85)^a^
Augmentation of labor	0.93 (0.88-0.98)^b^	0.89 (0.80-0.98)^b^	0.74 (0.48-1.13)
Type of labor			
Spontaneous	1 [Reference]	1 [Reference]	1 [Reference]
Induced	1.00 (0.94-1.07)	0.78 (0.70-0.87)^b^	0.51 (0.31-0.82)^b^
None	0.96 (0.86-1.07)	0.75 (0.60-0.93)^b^	1.93 (1.08-3.41)^a^
Unknown or missing	0.64 (0.51-0.80)^b^	0.60 (0.40-0.86)^b^	0
Maternal anesthesia			
Epidural	1.85 (1.75-1.96)^a^	1.58 (1.43-1.75)^a^	1.05 (0.68-1.60)
Spinal	1.42 (1.30-1.56)	1.04 (0.86-1.25)	0.72 (0.37-1.33)
General	4.77 (4.34-5.24)^a^	7.28 (6.18-8.55)^a^	15.94 (9.85-26.01)^a^
MSAF	2.26 (2.13-2.40)^a^	1.51 (1.35-1.69)^a^	2.35 (1.55-3.52)^a^
Fetal or neonatal factors			
GA in completed wk			
40	1 [Reference]	1 [Reference]	1 [Reference]
34	3.99 (3.42-4.65)^a^	2.61 (1.92-3.51)^a^	4.49 (1.77-10.94)^a^
35	2.77 (2.40-3.19)^a^	1.59 (1.17-2.12)^a^	1.41 (0.43-3.91)
36	1.85 (1.63-2.10)^a^	1.48 (1.17-1.85)^a^	1.54 (0.62-3.51)
37	1.48 (1.33-1.64)^a^	1.17 (0.97-1.41)	0.92 (0.39-1.98)
38	1.05 (1.33-1.64)^a^	0.96 (0.83-1.11)	0.84 (0.44-1.55)
39	1.03 (0.96-1.11)	0.94 (0.83-1.07)	0.88 (0.16-1.49)
41	1.15 (1.05-1.26)^a^	1.06 (0.91-1.24)	1.35 (0.74-2.41)
42	1.23 (0.79-1.83)	2.56 (1.55-3.96)^a^	1.92 (0.11-9.42)
43	3.95 (0.63-13.53)	0.00 (0.00-0.01)^b^	0
Multiple births	0.79 (0.69-0.90)^b^	0.50 (0.35-0.68)^b^	0.28 (0.07-0.83)^b^
Birth weight, per 1-kg increase	0.83 (0.78-0.88)^b^	0.76 (0.68-0.85)^b^	0.81 (0.53-1.22)
Macrosomia	1.87 (1.55-2.24)^a^	1.84 (1.31-2.52)^a^	1.99 (0.55-5.65)

Abbreviations: GA, gestational age; MDI, Material Deprivation Index; MSAF, meconium-stained amniotic fluid; OOH, out of hospital; OR, odds ratio; RI, Remoteness Index.

^a^ Statistically significantly higher OR.

^b^ Statistically significantly lower OR.

### Maternal SES

There were higher odds of ANRI for births in the less deprived quintile (OR, 1.17; 95% CI, 1.09-1.25) compared with the least deprived quintile due to higher odds of endotracheal intubation (OR, 1.12; 95% CI, 1.04-1.22) (Table 3). There were no other statistically significant differences by maternal SES.

### Remoteness of Maternal Residence

Neonates born to mothers living in less accessible areas had higher odds of receiving ANRI (OR, 1.30; 95% CI, 1.18-1.43) due to increased endotracheal intubation (OR, 1.45; 95% CI, 1.30-1.61) (Table 3). Neonates had lower odds of ANRI when born to mothers living in accessible areas (OR, 0.78; 95% CI, 0.73-0.83) and remote areas (OR, 0.79; 95% CI, 0.63-0.99) (Table 2; Figure 1). There were no statistically significant differences for births in very remote areas.

### Birth-Site Level of Service

Birth-site level of service was associated with all outcomes. Births at level 3 sites had lower odds of ANRI (OR, 0.57; 95% CI, 0.53-0.61) and all other interventions (Table 2; Figure 1). Births at level-1A sites had higher odds of ANRI (OR, 2.53; 95% CI, 1.99-3.15), lower odds of endotracheal intubation, and much higher odds for chest compressions (OR, 7.32; 95% CI, 5.61-9.40). Home births also had higher odds of ANRI (OR, 1.44; 95% CI, 1.18-1.74). Level-1B and level-1C sites were associated with lower odds of ANRI. Overall, level 1 sites had lower odds of endotracheal intubation (level 1C: OR, 0.54; 95% CI, 0.49-0.60; level 1B: OR, 0.52; 95% CI, 0.45-0.59; and level 1A: OR, 0.4 95% CI, 0.20-0.71) and higher odds of chest compressions. Home births also were associated with lower odds of endotracheal intubation (OR, 0.27; 95% CI, 0.16-0.43). Odds for chest compressions were highest for births at level-1A sites (OR, 7.32; 95% CI, 5.61-9.40), home (OR, 4.12; 95% CI, 3.29-5.11), and level 0 sites (OR, 2.86; 95% CI, 1.13-5.90) (Table 3). There were no statistically significant differences for unplanned out-of-hospital births.

### Clinical Factors

Clinical factors with the highest odds of any ANRI were maternal general anesthesia (OR, 4.89; 95% CI, 4.47-5.34), lower gestational age (dose response, highest OR at 34 weeks: 3.60; 95% CI, 3.11-4.15), MSAF (OR, 2.05; 95% CI, 1.94-2.17), macrosomia (OR, 1.83; 95% CI, 1.55-2.16), epidural anesthesia (OR, 1.82; 95% CI, 1.72-1.91), and any type of cesarean delivery (OR, 1.80; 95% CI, 1.60-2.02) (Table 2; Figure 2). Emergency cesarean delivery (OR, 1.45; 95% CI, 1.30-1.62) had higher odds of ANRI compared with elective cesarean delivery (OR, 0.78; 95% CI, 0.69-0.89). Maternal age younger than 20 years had the highest odds of ANRI (OR, 1.32; 95% CI, 1.18-1.47), while maternal age older than 35 years showed no difference.

General anesthesia was associated with higher odds of endotracheal intubation (OR, 4.77; 95% CI, 4.34-5.24), chest compressions (OR, 7.28; 95% CI, 6.18-8.55), and epinephrine administration (OR, 15.94; 95% CI, 9.85-26.01) (Table 3). Gestational age less than 37 weeks demonstrated a dose response for endotracheal intubation and chest compressions but not for epinephrine administration. Both MSAF and macrosomia had increased odds for endotracheal intubation (OR, 2.26 [95% CI, 2.13-2.40] and 1.87 [95% CI, 1.55-2.24]) and chest compressions (OR, 1.51 [95% CI, 1.35-1.69] and 1.84 [95% CI, 1.31-2.52]). For every 1-kg birth weight increase, there was a corresponding reduction in odds of ANRI (OR, 0.82; 95% CI, 0.78-0.87), endotracheal intubation (OR, 0.83; 95% CI, 0.78-0.88), and chest compressions (OR, 0.76; 95% CI, 0.68-0.85).

The only clinical factors associated with increased odds of epinephrine administration were maternal general anesthesia (OR, 15.94; 95% CI, 9.85-26.01), any type of cesarean delivery (OR, 5.51; 95% CI, 2.51-11.65), 34 weeks’ gestational age (OR, 4.49; 95% CI, 1.77-10.94), forceps or vacuum-assisted labor (OR, 4.35; 95% CI, 2.37-7.85), and no labor (OR, 1.93; 95% CI, 1.08-3.41).

### Missing Data

Sensitivity analysis results were consistent between approaches, suggesting that missing data had minimal implications for the results (eTables 3 and 4 in Supplement 1). For MSAF, which had the highest missing rate (18.6%), the difference in OR estimates between the analyses was less than 0.1 (eTable 2 in Supplement 1).

## Discussion

To our knowledge, this study is one of the largest investigations into the associations of clinical, SES, and health system factors with ANRIs in late preterm and term newborns. Our findings are as follows. First, in a model that included clinical factors, maternal SES and remoteness of residence were not associated with odds of ANRI. Second, birth-site level of service was associated with higher odds of ANRI and individual interventions (endotracheal intubation, chest compression, epinephrine administration) compared with maternal SES, maternal remoteness of residence, and some clinical factors. Third, the most significant clinical factors associated with ANRI were lower gestational age, cesarean delivery, maternal general anesthesia, MSAF, and macrosomia. Fourth, maternal general anesthesia was associated with increased odds of any ANRI, endotracheal intubation, chest compressions, and epinephrine administration.

### Maternal SES and Remoteness of Residence

We hypothesized that neonates born to mothers with lower SES would have higher ANRI odds, as lower maternal SES have been associated with preterm births and low birth weight.^6,29,30^ However, neither lower maternal SES nor remote residence was associated with increased odds of ANRI or individual interventions. Similarly, Ko et al^31^ reported that maternal residence in a low-income neighborhood or sparsely populated area was not associated with higher odds of mortality or major morbidities in extremely preterm infants admitted to Canadian NICUs. There are several possible explanations for this finding. First, lower maternal SES could have resulted in higher rates of risk factors such as prematurity and low birth weight. Therefore, in a multivariable model, odds attributed to lower SES were low. Second, Alberta’s universal health system might have mitigated the impact of lower maternal SES, as all persons could access public hospitals regardless of finances. Alternatively, neonates born to mothers with lower SES were not receiving ANRI due to lesser care access but rather due to lesser need. Third, mothers with lower SES could have had more stillbirths, which were not included.

Similarly, we hypothesized that neonates born to mothers living in remote areas would have higher ANRI odds due to less perinatal care access. This was not demonstrated in the results. However, there were few births from mothers living remotely. This finding, combined with low event rates, could mean that we lacked statistical power. Additionally, remote residents may have traveled for hospital births, thus reducing the implications of their location.

### Birth-Site Level of Service

There were differences attributed to birth-site level of service, despite standardized NRP training. First, births at level 3 hospitals had the lowest odds of all outcomes. This result was likely from optimal neonatal resuscitation due to training and experience, team composition, and equipment and processes for advanced resuscitations. Deliveries triaged to level 3 sites may also have had different obstetrical management.

Level-1B and level-1C births also had lower ANRI odds, compared with level 2 births, due to fewer endotracheal intubations. This outcome might be from appropriate obstetrical triaging and fewer endotracheal intubations due to limitations in health care practitioner skills. The higher odds of chest compressions in level-1B births may have been due to more newborns receiving inadequate ventilation or health care practitioners performing chest compressions before optimizing ventilation, using the adult cardiac–airway–breathing resuscitation approach. Our findings are congruent with those of a registry study of more than 1000 neonates in the US who received chest compressions at birth, 79% of whom had compressions initiated before intubation.^32^

Lowest level of service births had higher odds of ANRI, lower odds of endotracheal intubation, and higher odds of chest compressions. Possible explanations are (1) unpreparedness of lower-level facilities for emergency obstetrical and neonatal management, (2) reliance on adult cardiac–airway–breathing resuscitation practices, and (3) lack of comfort or capability with endotracheal intubations. These findings reflect a need to improve ventilation-focused neonatal resuscitation outside of tertiary centers.

### Clinical Risk Factors

Currently, the eighth edition of the NRP textbook lists 4 prebirth questions for health care practitioners to ask in preparation for neonatal resuscitation: (1) What is the expected gestational age? (2) Is the amniotic fluid clear? (3) Are there additional risk factors? (4) What is the umbilical cord management plan?^18^ In contrast, the European Resuscitation Council guidelines explicitly list antenatal and intrapartum risk factors, many of which were included in our study.^33^ Our study confirms that prematurity and MSAF convey risks for ANRI. In contrast, maternal factors, such as hypertension, diabetes, and prepregnancy weight, had a less than anticipated role in ANRI. This finding could be attributed to optimal obstetrical management or data limitations.

Furthermore, maternal general anesthesia was associated with higher odds of ANRI. This finding might be explained by the independent outcome of general anesthetic exposure in the fetus or neonate.^34,35^ First, maternal anesthesia could cause neonatal apnea. If adequate mask ventilation is not performed, the apneic neonate can develop hypoxemia leading to cardiac arrest. Second, general anesthesia could adversely affect fetal oxygenation and circulation, leading to perinatal asphyxia. Therefore, when general anesthesia is used, health care practitioners should be prepared for resuscitation. European Resuscitation Council guidelines already explicitly name general anesthesia as an intrapartum risk factor^33^; this should be further emphasized, and educational programs, such as the NRP, should consider adding maternal anesthetic method as an explicit prebirth question. Third, general anesthesia could be a marker of truly emergent cesarean delivery, where there is insufficient time for spinal or epidural anesthetic.

### Limitations

Our study had limitations. First, as an administrative data study, it was limited by the available population; thus, there were large uncertainties regarding remote populations and births at sites with lower levels of service, due to small numbers. Second, we were limited by data accuracy and completeness; some maternal factors (eg, smoking status and conditions other than hypertension or diabetes) could not be included as they were likely underreported. Furthermore, some clinical factors were reported by clinicians as *yes* or were left blank; thus, we cannot distinguish between no and missing data. Third, we could not differentiate between appropriate interventions and interventions resulting from inadequate prior steps or erroneous decision-making. Thus, our results reflect a combination of clinical need and practice variations, which can be difficult to untangle. However, births at level 3 sites had the lowest odds of any outcome, suggesting that optimal training and systems processes (ie, focused on steps before ANRI) rather than difference in clinical indications explain our findings.

## Conclusions

In this cross-sectional study of nearly 1 million late preterm and term neonates, maternal SES and maternal remoteness of residence were not associated with increased odds of ANRI. Birth sites with a lower level of service were associated with higher odds of chest compressions but lower odds of endotracheal intubations, highlighting a need to improve neonatal resuscitation in these settings. Health care practitioners should be prepared to resuscitate neonates exposed to maternal general anesthesia.

## Notes

Supplement 1. **eTable 1.** Univariable Level Logistic Regression Model **eTable 2.** Missing Data Rates **eFigure.** Heatmap of Primary and Secondary Outcomes **eTable 3.** Multivariable Logistics Regression Model and Sensitivity Analyses for Primary Outcome Any ANRI **eTable 4.** Comparison for Original Stepwise Variable Selection and Sensitivity Analyses for Primary Outcome of Any ANRI

Supplement 2.
Data Sharing Statement
