# Supplementary material for: Clinical and Nonclinical Factors and Advanced Neonatal Resuscitation Interventions
Source: JAMA Netw Open. 2026 Apr 30;9(4):e269923. doi: 10.1001/jamanetworkopen.2026.9923 (PMC13133688; doi:10.1001/jamanetworkopen.2026.9923)
Supplement: Supplement 1. — eTable 1. Univariable Level Logistic Regression Model eTable 2. Missing Data Rates eFigure. Heatmap of Primary and Secondary Outcomes eTable 3. Multivariable Logistics Regression Model and Sensitivity Analyses for Primary Outcome Any ANRI eTable 4. Comparison for Original Stepwise Variable Selection and Sensitivity Analyses for Primary Outcome of Any ANRI [file jamanetwopen-e269923-s001.pdf]

Supplemental Online Content

Pickett B, Pan B, Crawford S, et al. Clinical and nonclinical factors and advanced neonatal resuscitation interventions. *JAMA Netw Open*. 2026;9(4):e269923. doi:10.1001/jamanetworkopen.2026.9923

eTable 1. Univariable Level Logistic Regression Model

eTable 2. Missing Data Rates

eFigure. Heatmap of Primary and Secondary Outcomes

eTable 3. Multivariable Logistics Regression Model and Sensitivity Analyses for Primary Outcome Any ANRI

eTable 4. Comparison for Original Stepwise Variable Selection and Sensitivity Analyses for Primary Outcome of Any ANRI

This supplemental material has been provided by the authors to give readers additional information about their work.

© 2026 Pickett B et al. *JAMA Network Open*

eTable 1. Univariable Level Logistic Regression Model

Variable	Variable Categories	N	Univariate Odds Ratio (95 % CI)	P-value
**SES-HS Factors**				
**Pampalon Material Deprivation Index**	Least Deprived	165,422	Reference	-
	Less Deprived	181,015	1.17 (1.09 – 1.25)	<0.0001
	Moderately Deprived	178,658	1.10 (1.03 – 1.18)	0.0072
	More Deprived	178,003	1.10 (1.02 – 1.17)	0.011
	Most Deprived	214,600	0.97 (0.91 – 1.04)	0.45
**Remoteness Groups K-Clustering**	Easily Accessible Area	702,274	Reference	-
	Accessible Area	180277	0.79 (0.74 – 0.83)	<0.0001
	Less Accessible Area	57,066	1.13 (1.04 – 1.22)	0.0042
	Remote Area	15,406	0.68 (0.56 – 0.82)	<0.0001
	Very Remote Area	1,510	0.73 (0.38 – 1.26)	0.31
**Birth Site Level of Service**	Level 2	547,426	Reference	-
	Level 0	1,307	0.94 (0.52 – 1.56)	0.83
	Level 1A	7,690	1.22 (1.00 – 1.49)	0.048
	Level 1B	93,735	0.70 (0.64 – 0.75)	<0.0001
	Level 1C	93,585	0.68 (0.63 – 0.74)	<0.0001
	Level 3	208,264	0.83 (0.79 – 0.88)	<0.0001
	Unplanned out of hospital births	276	1.73 (0.62 – 3.76)	0.22
	Midwifery Planned Home Birth	14,182	0.77 (0.64 – 0.93)	0.0065
**Maternal Factors**				
**Maternal age in Years**			1.00 (0.99 – 1.00)	0.11
**Maternal Age <20**		37,364	1.29 (1.17 – 1.41)	<0.0001
**Maternal Age ≥35**		171,468	1.03 (0.98 – 1.09)	0.22
**Maternal Age Categories**	<20 years	37,464	1.30 (1.18 – 1.43)	<0.0001
	20 – 35 years	800,996	Reference	-
	> 35 years	128,015	1.08 (1.02 – 1.14)	0.014
**Heart Disease - Asymptomatic**		4516	1.05 (0.77 – 1.39)	0.76
**Heart Disease - Symptomatic**		684	0.61 (0.19 – 1.43)	0.33
**Pre-existing Hypertension**		8924	2.09 (1.79 – 2.42)	<0.0001
**Gestational Hypertension**		52,437	1.93 (1.80 – 2.06)	<0.0001
**Pre-pregnancy Weight >91kg**		88,039	1.38 (1.30 – 1.47)	<0.0001
**Pre-existing Diabetes**		10,911	1.90 (1.64 – 2.19)	<0.0001
**Gestational Diabetes**		57,358	1.26 (1.17 – 1.36)	<0.0001
**Previous C-sections**		139,199	0.84 (0.78 – 0.89)	<0.0001
**Intrapartum Factors**				
**C-section - Elective**		142,892	0.84 (0.79 – 0.89)	<0.0001
**C-section - Emergency**		87,731	3.54 (3.37 – 3.71)	<0.0001
**C-section – all types**		264,252	2.08 (2.00 – 2.17)	<0.0001
**Induction of Labour**		290,076	1.26 (1.20 – 1.31)	<0.0001
**Forceps or Vacuum**		115,168	1.12 (1.054 – 1.19)	0.0002
**Augmentation**		419,307	0.92 (0.89 – 0.96)	0.0002
**Type of Labour**	Spontaneous	541,342	Reference	-
	Induced	290,076	1.27 (1.22 – 1.33)	<0.0001
	None	117,960	1.09 (1.02 – 1.16)	0.011
	Unknown or missing	17,097	0.84 (0.70 – 1.00)	0.060
**Anesthetic Method – Epidural**		290,856	1.77 (1.70 – 1.84)	<0.0001
**Anesthetic Method – General Anesthesia**		13,350	7.29 (6.77 – 7.85)	<0.0001
**Anesthetic Method - Spinal**		89,066	1.49 (1.40 – 1.58)	<0.0001
**Meconium-Stained Amniotic Fluid**		111,535	2.07 (1.97 – 2.17)	<0.0001
**Fetal / Neonatal Factors**				
**Gestational Age at Delivery (completed weeks)**			0.86 (0.85 – 0.88)	<0.0001
**Multiple Births**		26,057	1.61 (1.45 – 1.78)	<0.0001
**Preterm (< 37 weeks)**		62,416	2.65 (2.50 – 2.81)	<0.0001
**Low Birth Weight**		45,253	2.80 (2.63 – 2.98)	<0.0001
**Birth Weight (kg)**			0.67 (0.64 – 0.69)	<0.0001
**Large for Gestational Age**		63,788	1.19 (1.11 – 1.27)	<0.0001
**Macrosomia**		14,771	1.59 (1.39 – 1.82)	<0.0001
**Small for Gestational age**		93,091	1.65 (1.55 – 1.74)	<0.0001
**Extreme Large for Gestational Age**		26,979	1.47 (1.32 – 1.63)	<0.0001
**Congenital Anomaly**		19,096	2.72 (2.37 – 3.11)	<0.0001

© 2026 Pickett B et al. *JAMA Network Open*

Variables excluded from Multi-variable Logistics Regression

- Excluded for more inclusive variables:
  - Low birth weight
  - Preterm
- Other exclusions:
  - Maternal Heart Disease – Symptomatic: excluded as it caused numerical issues by drastically changing the results

Variables excluded after Stepwise Variable Selection

- Infant Variables
  - Small for gestational age
  - Large for gestational age
  - Extremely large for gestational age
  - Congenital anomalies
- Maternal Variables
  - Heart disease – asymptomatic
  - Gestational Diabetes

© 2026 Pickett B et al. *JAMA Network Open*

eTable 2. Missing Data Rates

Variable Group	Variable / Categories		Missing (%)
**Material deprivation index**	Least Deprived		5.1
	Less Deprived		
	Moderately Deprived		
	More Deprived		
	Most Deprived		
**Maternal Remoteness of Residence**	Easily Accessible Area		1.0
	Accessible Area		
	Less Accessible Area		
	Remote Area		
	Very Remote Area		
**Birth Hospital Level of Service**	Level 2		0
	Level 0		
	Level 1A		
	Level 1B		
	Level 1C		
	Level 3		
	Unplanned Out of Hospital Birth		
	Midwifery Planned Home Birth		
**Maternal Factors**	Maternal Age	<20 years	0
		20 – 35 years	
		> 35 years	
	Pre-existing Hypertension		0.9
	Gestational Hypertension		0.8
	Pre-pregnancy Weight >91kg		0.8
	Pre-existing Diabetes		0.8
	Previous C-sections		0.8
**Intrapartum Factors**	C-section (any type)		0
	Elective C-section		0.9
	Emergency C-section		0
	Forceps or Vacuum Assisted		0
	Augmentation		0
	Type of Labour	Spontaneous	1.8
		Induced	
		None	
		Unknown or missing	
	Epidural Anesthesia		0
	Spinal Anesthesia		0
	General Anesthesia		0
	Meconium-Stained Amniotic Fluid		18.6
**Infant Factors**	Gestational Age at Birth	40	0
		34	
		35	
		36	
		37	
		38	
		39	
		41	
		42	
		43	
		44	
		45	
	Multiple Births		0
	Birth Weight (per 1kg increase)		0
	Macrosomia		0

© 2026 Pickett B et al. *JAMA Network Open*

eFigure. Heatmap of Primary and Secondary Outcomes

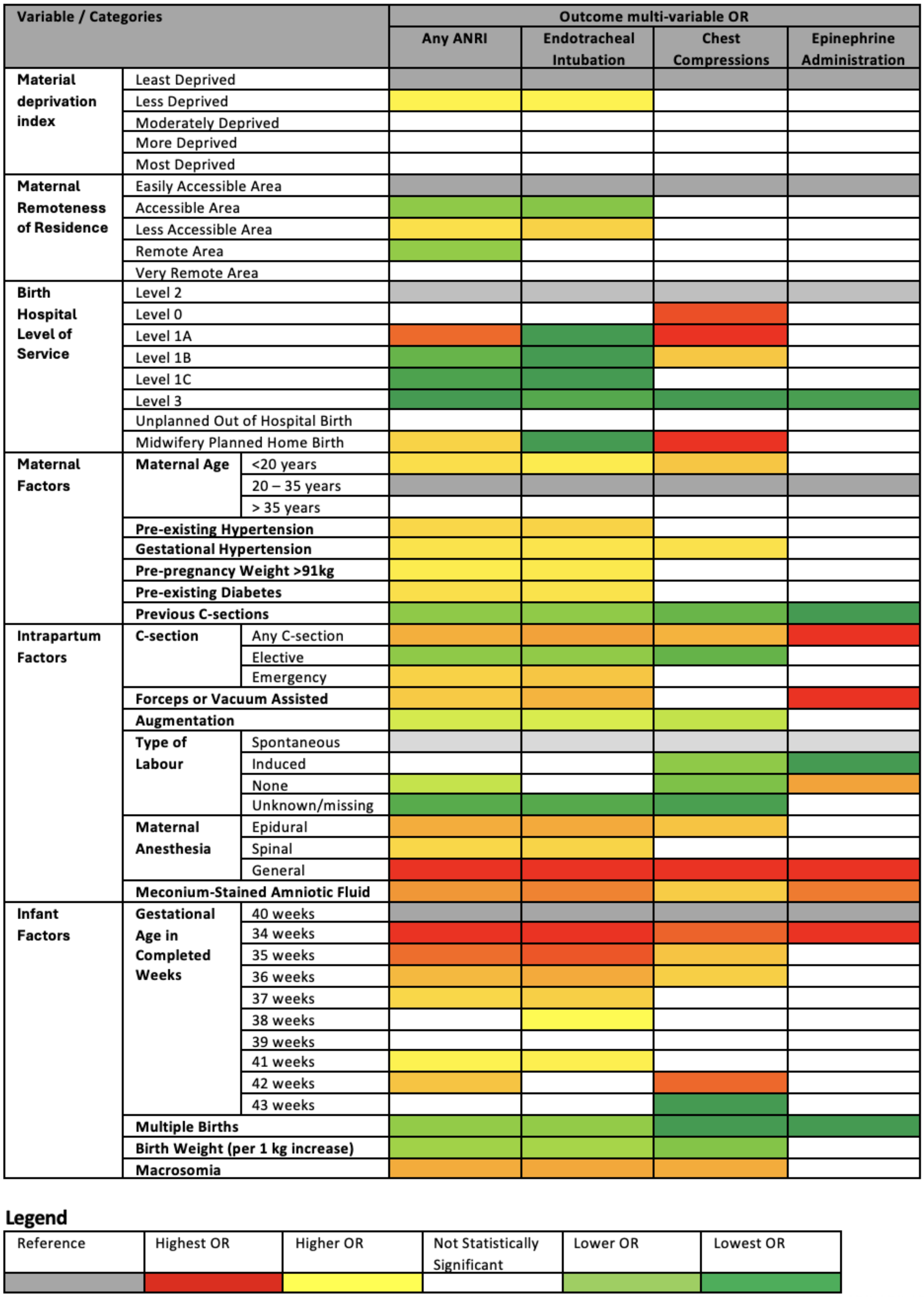

© 2026 Pickett B et al. *JAMA Network Open*

eTable 3. Multivariable Logistics Regression Model and Sensitivity Analyses for Primary Outcome Any ANRI

Clinical and Non-Clinical Variables		Multivariable Odds Ratio (95% Confidence Interval)		
**Variable**	**Variable Categories**	**Original Analysis Any ANRI**	**Resuscitation measures Listed as Unknown Treated Excluded**	**Data Imputed for all Missing Data***
**SES-HS Factors**				
**Pampalon Material Deprivation Index**	Least Deprived	Reference	Reference	Reference
	Less Deprived	1.12 (1.04 – 1.21)	1.12 (1.04 – 1.21)	1.10 (1.03 – 1.18)
	Moderately Deprived	1.04 (0.97 – 1.13)	1.04 (0.96 – 1.12)	1.06 (0.99 – 1.13)
	More Deprived	1.04 (0.96 – 1.12)	1.03 (0.96 – 1.12)	1.04 (0.97 – 1.13)
	Most Deprived	0.95 (0.88 – 1.02)	0.96 (0.89 – 1.03)	0.98 (0.92 – 1.05)
**Remoteness Groups K-Clustering**	Easily Accessible Area	Reference	Reference	Reference
	Accessible Area	0.78 (0.73 – 0.83)	0.78 (0.73 – 0.83)	0.81 (0.76 – 0.86)
	Less Accessible Area	1.30 (1.18 – 1.43)	1.31 (1.18 – 1.44)	1.23 (1.12 – 1.34)
	Remote Area	0.79 (0.63 – 0.99)	0.84 (0.66 – 1.04)	0.86 (0.70 – 1.03)
	Very Remote Area	0.65 (0.20 – 1.55)	0.75 (0.23 – 1.82)	0.90 (0.50 – 1.48)
**Birth Site Level of Service**	Level 2	Reference	Reference	Reference
	Level 0	1.37 (0.70 – 2.37)	1.37 (0.70 – 2.36)	1.43 (0.78 – 2.37)
	Level 1A	2.53 (2.0 – 3.15)	2.50 (1.98 – 3.12)	2.37 (1.93 – 2.89)
	Level 1B	0.69 (0.62 – 0.77)	0.73 (0.65 – 0.81)	0.83 (0.76 – 0.91)
	Level 1C	0.61 (0.56 – 0.67)	0.62 (0.57 – 0.68)	0.68 (0.63 – 0.74)
	Level 3	0.57 (0.53 – 0.61)	0.72 (0.67 – 0.76)	0.87 (0.83 – 0.92)
	Unplanned out of hospital births	2.25 (0.56 – 5.94)	2.85 (0.70 – 7.56)	3.52 (1.39 – 7.25)
	Midwifery Planned Home Birth	1.44 (1.18 – 1.74)	1.45 (1.19 – 1.75)	1.57 (1.29 – 1.88)
**Maternal Factors**				
**Maternal Age Categories**	<20 years	1.32 (1.18 – 1.47)	1.29 (1.16 – 1.44)	1.28 (1.16 - 1.40)
	20 – 35 years	Reference	Reference	Reference
	> 35 years	0.99 (0.93 – 1.06)	0.99 (0.93 – 1.06)	1.02 (0.96 – 1.08)
**Heart Disease - Asymptomatic**		0.99 (0.69 – 1.37)	1.0 (0.70 – 1.38)	1.05 (0.79 – 1.37)
**Pre-existing Hypertension**		1.40 (1.18 – 1.65)	1.41 (1.19 – 1.66)	1.32 (1.14 – 1.53)
**Gestational Hypertension**		1.27 (1.16 – 1.37)	1.26 (1.16 – 1.37)	1.30 (1.21 – 1.40)
**Pre-pregnancy Weight >91kg**		1.18 (1.10 – 1.27)	1.18 (1.09 – 1.26)	1.18 (1.11 – 1.26)
**Pre-existing Diabetes**		1.31 (1.11 – 1.53)	1.29 (1.10 – 1.52)	1.38 (1.20 – 1.59)
**Gestational Diabetes**		1.04 (0.95 – 1.13)	1.06 (0.97 – 1.15)	1.07 (0.99 – 1.16)
**Previous C-sections**		0.78 (0.72 – 0.84)	0.79 (0.73 – 0.85)	0.77 (0.72 – 0.83)
**Intrapartum Factors**				
**C-section - Elective**		0.78 (0.69 – 0.89)	0.78 (0.69 – 0.88)	0.79 (0.71 – 0.87)
**C-section - Emergency**		1.45 (1.30 – 1.62)	1.48 (1.32 – 1.65)	1.46 (1.34 – 1.61)
**C-section – all types**		1.80 (1.60 – 2.02)	1.73 (1.54 – 1.94)	1.78 (1.62 – 1.95)
**Forceps or Vacuum**		1.52 (1.41 – 1.63)	1.48 (1.38 – 1.59)	1.43 (1.34 – 1.53)
**Augmentation**		0.96 (0.87 – 0.96)	0.92 (0.87 – 0.97)	0.91 (0.87 – 0.96)
**Type of Labour**	Spontaneous	Reference	Reference	Reference
	Induced	0.96 (0.91 – 1.02)	0.967 (0.91 – 1.02)	0.96 (0.91 – 1.00)
	None	0.88 (0.80 – 0.98)	0.90 (0.82 – 1.00)	0.85 (0.78 – 0.93)
	Unknown or missing	0.65 (0.53 – 0.79)	0.61 (0.50 – 0.75)	0.64 (0.53 – 0.77)
**Anesthetic Method – Epidural**		1.81 (1.72 – 1.91)	1.82 (1.73 – 1.92)	1.95 (1.86 – 2.04)
**Anesthetic Method – General Anesthesia**		4.89 (1.47 – 5.34)	4.72 (4.32 – 5.16)	4.81 (4.44 – 5.21)
**Anesthetic Method - Spinal**		1.39 (1.27 – 1.51)	1.40 (1.29 – 1.53)	1.51 (1.40 – 1.62)
**Meconium-Stained Amniotic Fluid**		2.05 (1.94 – 2.17)	1.99 (1.89 – 2.11)	2.01 (1.91 – 2.11)
**Fetal / Neonatal Factors**				
**Gestational Age at Delivery (completed weeks)**	40	Reference	Reference	Reference
	34	3.59 (3.10 – 4.14)	3.46 (2.99 – 4.0)	3.37 (2.97 – 3.82)
	35	2.48 (2.17 – 2.84)	2.42 (2.12 – 2.77)	2.33 (2.07 – 2.63)
	36	1.70 (1.51 – 1.91)	1.68 (1.49 – 1.88)	1.62 (1.46 – 1.80)
	37	1.38 (1.26 – 1.52)	1.39 (1.26 – 1.52)	1.28 (1.18 – 1.40)
	38	1.02 (0.94 – 1.10)	1.02 (0.94 – 1.10)	1.01 (0.94 – 1.08)
	39	1.02 (0.95 – 1.09)	1.02 (0.96 – 1.09)	1.00 (0.94 – 1.06)
	41	1.13 (1.04 – 1.22)	1.12 (1.03 – 1.21)	1.17 (1.09 – 1.25)
	42	1.58 (1.11 – 2.17)	1.56 (1.10 – 2.15)	1.47 (1.08 – 1.94)
	43	3.19 (0.51 – 10.71)	2.99 (0.48 – 10.04)	3.21 (0.78 – 8.79)
**Multiple Births**		0.79 (0.69 – 0.89)	0.80 (0.70 – 0.91)	0.80 (0.71 – 0.89)
**Birth Weight (per 1 kg)**		0.82 (0.78 – 0.87)	0.81 (0.77 – 0.86)	0.79 (0.75 – 0.82)
**Macrosomia**		1.83 (1.54 – 2.16)	1.84 (1.55 – 2.17)	1.90 (1.63 – 2.20)

* Missing data were imputed using multiple imputation with 5 imputed datasets (m = 5), applying the Predictive Mean Matching method.

© 2026 Pickett B et al. *JAMA Network Open*

eTable 4. Comparison for Original Stepwise Variable Selection and Sensitivity Analyses for Primary Outcome of Any ANRI

Clinical and Non-Clinical Variables		Multivariable Odds Ratio (95% Confidence Interval)		
**Variable**	**Variable Categories**	**Original Analysis For any ANRI**	**Resuscitation measures Listed as Unknown Treated Excluded**	**Data Imputed for all Missing Data**
**SES-HS Factors**				
**Pampalon Material Deprivation Index**	Least Deprived	Reference	Reference	Reference
	Less Deprived	1.12 (1.04 – 1.21)	1.12 (1.04 – 1.20)	1.10 (1.03 – 1.18)
	Moderately Deprived	1.04 (0.97 – 1.13)	1.04 (0.96 – 1.12)	1.06 (0.99 – 1.13)
	More Deprived	1.04 (0.96 – 1.12)	1.03 (0.96 – 1.12)	1.04 (0.97 – 1.11)
	Most Deprived	0.95 (0.88 – 1.02)	0.96 (0.89 – 1.03)	0.98 (0.92 – 1.05)
**Remoteness Groups K-Clustering**	Easily Accessible Area	Reference	Reference	Reference
	Accessible Area	0.78 (0.73 – 0.83)	0.78 (0.73 – 0.83)	0.81 (0.76 – 0.86)
	Less Accessible Area	1.30 (1.18 – 1.43)	1.31 (1.18 – 1.44)	1.23 (1.12 – 1.34)
	Remote Area	0.79 (0.63 – 0.99)	0.84 (0.66 – 1.04)	0.86 (0.70 – 1.03)
	Very Remote Area	0.65 (0.20 – 1.55)	0.75 (0.23 – 1.82)	0.90 (0.50 – 1.48)
**Birth Site Level of Service**	Level 2	Reference	Reference	Reference
	Level 0	1.36 (0.71 – 2.37)	1.37 (0.70 – 2.36)	1.43 (0.78 – 2.37)
	Level 1A	2.53 (1.99 – 3.15)	2.50 (1.97 – 3.12)	2.37 (1.93 – 2.89)
	Level 1B	0.69 (0.62 – 0.77)	0.73 (0.65 – 0.81)	0.82 (0.76 – 0.91)
	Level 1C	0.61 (0.56 – 0.66)	0.62 (0.57 – 0.68)	0.68 (0.63 – 0.74)
	Level 3	0.57 (0.53 – 0.61)	0.72 (0.67 – 0.76)	0.87 (0.83 – 0.92)
	Unplanned out of hospital births	2.25 (0.55 – 5.93)	2.85 (0.70 – 7.55)	3.52 (1.39 – 7.25)
	Midwifery Planned Home Birth	1.44 (1.18 – 1.74)	1.45 (1.18 – 1.75)	1.57 (1.29 – 1.88)
**Maternal Factors**				
**Maternal Age Categories**	<20 years	1.52 (1.26 – 1.82)	1.50 (1.25 – 1.80)	1.41 (1.19 – 1.65)
	20 – 35 years	Reference	Reference	Reference
	> 35 years	0.89 (0.77 – 1.02)	0.89 (0.77 – 1.02)	0.89 (0.79 – 1.01)
**Heart Disease - Asymptomatic**		Not selected	Not selected	Not selected
**Pre-existing Hypertension**		1.40 (1.18 – 1.65)	1.41 (1.19 – 1.66)	1.33 (1.14 – 1.54)
**Gestational Hypertension**		1.27 (1.17 – 1.37)	1.26 (1.16 - 1.37)	1.30 (1.21 – 1.40)
**Pre-pregnancy Weight >91kg**		1.19 (1.10 – 1.27)	1.18 (1.10 – 1.27)	1.18 (1.11 – 1.26)
**Pre-existing Diabetes**		1.31 (1.11 – 1.54)	1.31 (1.11 – 1.53)	1.38 (1.20 – 1.59)
**Gestational Diabetes**		Not selected	Not selected	1.07 (0.99 – 1.16)
**Previous C-sections**		0.78 (0.72 – 0.84)	0.79 (0.73 – 0.85)	0.77 (0.72 – 0.83)
**Intrapartum Factors**				
**C-section - Elective**		0.78 (0.69 – 0.89)	0.78 (0.69 – 0.88)	0.79 (0.72 – 0.87)
**C-section - Emergency**		1.45 (1.30 – 1.62)	1.48 (1.32 – 1.65)	1.46 (1.34 – 1.61)
**C-section – all types**		1.80 (1.60 – 2.02)	1.73 (1.54 – 1.94)	1.78 (1.62 – 1.95)
**Forceps or Vacuum**		1.52 (1.41 – 1.63)	1.48 (1.38 – 1.59)	1.43 (1.34 – 1.53)
**Augmentation**		0.92 (0.87 – 0.97)	0.92 (0.88 – 0.97)	0.91 (0.87 – 0.96)
**Type of Labour**	Spontaneous	Reference	Reference	Reference
	Induced	0.96 (0.91 – 1.02)	0.97 (0.92 – 1.03)	0.96 (0.91 – 1.00)
	None	0.89 (0.80 – 0.98)	0.91 (0.82 – 1.00)	0.85 (0.78 – 0.93)
	Unknown or missing	0.65 (0.53 – 0.79)	0.61 (0.50 – 0.75)	0.64 (0.53 – 0.77)
**Anesthetic Method – Epidural**		1.82 (1.72 – 1.91)	1.82 (1.73 – 1.92)	1.95 (1.86 – 2.04)
**Anesthetic Method – General Anesthesia**		4.89 (4.47 – 5.34)	4.72 (4.31 – 5.16)	4.81 (4.44 – 5.21)
**Anesthetic Method - Spinal**		1.39 (1.27 – 1.51)	1.40 (1.29 – 1.53)	1.51 (1.40 – 1.62)
**Meconium-Stained Amniotic Fluid**		2.05 (1.94 – 2.17)	1.99 (1.89 – 2.11)	2.01 (1.91 – 2.11)
**Fetal / Neonatal Factors**				
**Gestational Age at Delivery (completed weeks)**	40	Reference	Reference	Reference
	34	3.60 (3.11 – 4.15)	3.48 (3.01 – 4.01)	3.37 (2.97 – 3.82)
	35	2.49 (2.18 – 2.85)	2.43 (2.12 – 2.78)	2.33 (2.07 – 2.63)
	36	1.70 (1.51 – 1.91)	1.68 (1.49 – 1.89)	1.62 (1.46 – 1.80)
	37	1.39 (1.26 – 1.53)	1.39 (1.26 – 1.83)	1.28 (1.18 – 1.40)
	38	1.02 (0.94 – 1.10)	1.02 (0.95 – 1.11)	1.01 (0.94 – 1.08)
	39	1.02 (0.95 – 1.09)	1.03 (0.96 – 1.10)	1.00 (0.94 – 1.07)
	41	1.12 (1.04 – 1.22)	1.12 (1.03 – 1.21)	1.16 (1.09 - 1.25)
	42	1.58 (1.11 – 2.17)	1.56 (1.10 – 2.14)	1.47 (1.08 – 1.94)
	43	3.19 (0.51 – 10.71)	2.99 (0.48 – 10.04)	3.21 (0.78 – 8.79)
**Multiple Births**		0.79 (0.69 – 0.89)	0.80 (0.70 – 0.90)	0.80 (0.71 – 0.89)
**Birth Weight (per 1 kg)**		0.82 (0.78 – 0.87)	0.81 (0.77 – 0.86)	0.79 (0.75 – 0.82)
**Macrosomia**		1.83 (1.55 – 2.16)	1.84 (1.55 – 2.17)	1.90 (1.63 – 2.20)

* Missing data were imputed using multiple imputation with 5 imputed datasets (m = 5), applying the Predictive Mean Matching method

© 2026 Pickett B et al. *JAMA Network Open*
