# Supplementary material for: Clinical and Nonclinical Factors and Advanced Neonatal Resuscitation Interventions
Source: JAMA Netw Open. 2026 Apr 30;9(4):e269923. doi: 10.1001/jamanetworkopen.2026.9923 (PMC13133688; doi:10.1001/jamanetworkopen.2026.9923)
Supplement: Supplement 2. — Data Sharing Statement [file jamanetwopen-e269923-s002.pdf]

Data Sharing Statement

Pickett. Clinical and Nonclinical Factors and Advanced Neonatal Resuscitation Interventions. *JAMA Netw Open*. Published April 30, 2026. doi:10.1001/jamanetworkopen.2026.9923

Data
Data available: No
